# ﻿Taxonomic study on the subgenus *Orientostichus*, the *Pterostichuspulcher* species group (Coleoptera, Carabidae, Pterostichus)

**DOI:** 10.3897/zookeys.1175.107636

**Published:** 2023-08-16

**Authors:** Wenqi Yin, Pingzhou Zhu, Hongliang Shi

**Affiliations:** 1 College of Forestry, Beijing Forestry University, Beijing 100083, China Beijing Forestry University Beijing China; 2 Department of Entomology, College of Plant Protection, China Agricultural University, Beijing 100093, China China Agricultural University Beijing China

**Keywords:** China, endophallus, male genitalia, new species, *Pterostichus*, Sichuan province

## Abstract

The *Pterostichuspulcher* species group of the subgenus Orientostichus Sciaky & Allegro is defined for *P.pulcher* Sciaky & Allegro and six new allied species. All seven species of this group are revised on morphological characters. Six new species are described from south of Sichuan province, China: *P.pemphis***sp. nov.** (type locality: Shuihaizi, Puge county, 27.33°N, 102.45°E), *P.orbicollis***sp. nov.** (Longzhoushan, Huili county, 26.79°N, 102.20°E), *P.leo***sp. nov.** (Shizishan, Jinyang county, 27.88°N, 103.23°E), *P.liyuani***sp. nov.** (Luojishan, Puge county, 27.58°N, 102.39°E), *P.condylus***sp. nov.** (Yele, Mianning county, 28.96°N, 102.16°E), *P.jialini***sp. nov.** (Jiamashi, Huidong county, 26.81°N, 102.68°E). *Tritrichischinensis* Jedlička, **syn. nov.**, a species previously misplaced in the subgenus Orientostichus, is excluded from the genus *Pterostichus* and confirmed to be a junior synonym of *Synuchusnitidusreticulatus* Lindroth, 1956.

## ﻿Introduction

The subgenus Orientostichus Sciaky & Allegro, 2013 includes a group of pterostichine beetles with a robust body, continuous marginal umbilicate pore series of elytra, and longitudinally twisted median lobe apex of the male genitalia. Some members of this subgenus have been placed in the *prattii* group (Jedlička 1962). Sciaky and Allegro (2013) erected the subgenus Orientostichus based on shared external and genital features and assigned fourteen species to it, among which 11 are distributed in China. Since then, no new species were described in this subgenus except *Tritrichischinensis* Jedlička, 1962, which was proposed to be included in this subgenus by Fedorenko (2018).

During our field investigations in the mountains of southwest China, it is found that the species richness of the subgenus Orientostichus far exceeded that which was known, and many undescribed species are narrowly distributed but locally abundant. As the first part of our revision to this subgenus, the present paper focuses on a small group mainly distributed in the Liangshan Yi Autonomous Prefecture in southern Sichuan, which is clearly distinguished from other members of the subgenus by their modified elytral intervals 3 and 5, and asetose endophallus with deeply grooved basal sclerotized projection. Thus, the main aim of the present study is to provide a taxonomic revision of the *Pterostichuspulcher* species group as defined herein, with descriptions of six new species, a key to all known seven species, and external features and male genital illustrations of all species. Additionally, the doubtful species *Tritrichischinensis* Jedlička which was previously assigned to this subgenus (Fedorenko 2018), is confirmed to be a junior synonym of *Synuchusnitidusreticulatus* Lindroth, and a brief discussion is provided.

## ﻿Material and method

The present study mainly bases on the examinations of specimens from Liangshan Yi Autonomous Prefecture, southern part of Sichuan province, China. Unless specified, specimens examined, including types of new species, are deposited in the collections of the Institute of Zoology, Chinese Academy of Sciences, Beijing, China (**IZAS**). Other collections cited in the present paper are indicated by the following abbreviations:

**CHYL** Collection of Haoyuan Li, Beijing, China;

**CYHL** Collection of Yihang Li, Beijing, China;

**NMPC**Národní Muzeum Přírodovědecké Muzeum, Prague, Czech Republic;

**ZMC** Zoologisk Museum of Copenhagen, Copenhagen, Denmark.

The body length (**BL**) was measured from the apical margin of the labrum to the elytral apex; body width (**BW**) was measured along the elytral greatest width. The pronotum width (**PW**) was measured along its greatest width; pronotum length (**PL**) was measured along its median line; basal width (**PBW**) was measured along its posterior margin. The apical lamella of the aedeagus was measured in dorsal view: length of apical lamella (**LL**) was the distance between the extreme apex and apical margin of the apical orifice; width of apical lamella (**LW**) was its basal width along apical margin of the apical orifice.

The terminology of the male genitalia and female genitalia follows Shi and Liang (2015). Other terms used, dissection techniques, endophallus everting procedures, and photography follow Shi et al. (2013). The abbreviations used in the endophallus and female genitalia are as follows: gonopore (**gp**), gonopore lobe (**gpl**), ventral basal lobe (**vb**), basal sclerotized projection (**bsp**), gonocoxite 1 (**g1**), gonocoxite 2 (**g2**), ensiform setae (**es**), nematiform setae (**ns**), bursa copulatrix (**bc**), common oviduct (**co**), seminal canal (**sc**), receptaculum (**rc**), spermathecal gland (**sg**), and spermathecal canal (**spc**).

## ﻿Taxonomic account

### ﻿Subgenus Orientostichus

Taxon classificationAnimaliaColeopteraCarabidae

Sciaky & Allegro, 2013

2EA92D77-CCEC-52E3-AFCB-BC6E66589349

Orientostichus Sciaky & Allegro, 2013: 113; Fedorenko 2018: 111.

#### Type species.

*Pterostichusprattii* Bates, 1890 (type locality: Wa-Shan, Sichuan, China).

#### Subgeneric characters.

Body moderately to strongly robust, medium to large sized, body length 13–32 mm. Terminal labial palpomere more or less expanded, usually subtriangular. Mesofemora with two setae on posterior ventral margin; metacoxae with two setae; metatrochanters without seta; fifth tarsomere usually setose ventrally. Elytral epipleura crossed. Umbilicate pore series on interval 9 continuous in middle (middle pores only slightly sparser than those near base and apex). Male sternite VII with or without secondary sexual modification. Male genitalia stout, apical lamella relatively long and twisted longitudinally. Endophallus with sclerotized projections on the ventral surface.

#### Taxonomic comments.

When erected, 14 species were included in the subgenus (Sciaky and Allegro 2013). Later, *Tritrichischinensis* Jedlička, 1962 was added to this subgenus as well (Fedorenko 2018: 111). However, this species actually belongs in the genus *Synuchus* Gyllenhal, 1810 (see below for details).

The monophyly of the subgenus Orientostichus is supported by its distinctive features of the male genitalia, namely the apical lamella more or less twisted longitudinally. The relationships among the 14 previously described species are not understood, because most of these species have few taxonomic characteristics useful to recognize them except for the diverse secondary modifications on male sternite VII. Nevertheless, the relationship of *P.pulcher* Sciaky & Allegro with another six new species described herein is supported by their morphological similarity in several features. We define the *Pterostichuspulcher* species group for the species of subgenus Orientostichus to include those species having the elytral intervals 3 and 5 each with ≥ 3 large and foveate discal pores, interrupting intervals and forming irregularly catenulate sculpturing. Affinities of the seven species belonging to the *P.pulcher* species group can be supported by characteristics of the endophallus of male genitalia: endophallus asetose, basal sclerotized projection (bsp) deeply grooved in the middle, forming a spiral-shaped or U-shaped structure (Figs 19–28). In contrast, in *Pterostichusprattii* and its related species, there are coarse setae on the dorsal-apical surface of endophallus (Fig. 29). Whereas in *Pterostichuscurtatus*, *P.perlutus* and some undescribed species related to them, such setae are absent, and the basal sclerotized projection is not grooved but slightly hooked (Fig. 30). These characteristic clearly distinguished them from the *P.pulcher* group species.

#### Morphological characters of the *Pterostichuspulcher* species group.

Relatively robust pterostichine beetles, medium-sized for the subgenus Orientostichus, BL 14.0–18.5 mm, BW 4.9–6.2 mm. Dorsal surface nearly black or a little brownish black, without evident metallic luster; mouthparts and tarsomeres sometimes reddish brown. Head medium-sized, nearly smooth. Eyes large and convex; two supraorbital setae present; frontal grooves deep, slightly sinuate, reaching mid-point of eyes; temporae short, slightly convex. Antennae exceeding pronotal base in one or two segments, antennomere 3 with or without accessory setae. Labrum and clypeus shallowly curved inward apically; mandibles straight and elongate, apex evenly curved; terminal labial and maxillary palpomere slightly expanded, subtriangular, a little more broadly expanded in males; penultimate labial palpomere with two setae along inner margin, without extra seta near apex; submentum with one lateral seta on each side. Pronotum circular or subcordate, disc with faint isodiametric microsculpture; widest near 1/3, with ≥ 1 mid-lateral setae; basal seta very close to posterior angle. Anterior margin markedly emarginate, narrowly bordered along its entire length. Anterior angles narrowly rounded, their apices not or slightly projected. Posterior margin almost straight, sometimes very shallowly concave in middle, much narrower than base of elytra between humeral angles. Disc moderately convex, often with transverse wrinkles aside median line. Basal foveae with inner and outer grooves well-defined but both without clear limits, these partly fused at base, outer groove evidently shorter than inner one. Elytra oblong, width a little greater than 1/2 of length, widest a little behind middle, with distinct isodiametric microsculpture in both males and females. Shoulders rounded, humeral angles obtuse and not projected outward, apex not dentate. Striae deep and impunctate, parascutellar pore usually absent, rarely present on base of stria 1; intervals clearly convex, elytral intervals 3 and 5 each with ≥ 3 large and foveate discal pores, sometimes discal pores also present on interval 7; these intervals are interrupted by discal pores, forming irregularly catenulate sculpturing. Umbilicate pore series on interval 9 composed of 21–23 pores, continuous in middle (middle pores only slightly sparser than those near base and apex, thus the basal, middle, and apical groups of pores cannot be clearly separated). Ventral side: Metepisternum length slightly shorter than basal width; sternite VII with one seta on each side in males, two in females; male sternite VII usually with secondary sexual modification. Fifth tarsomere setose ventrally. Male genitalia: Median lobe of aedeagus stout, curved near basal 1/3; apical lamella twisted longitudinally. Right paramere relatively short and stout, apex rounded. Endophallus strongly directed ventrally, with three sclerotized projections on ventral surface: two preapical projections very close to gonopore, small and slightly hooked; basal sclerotized projection (bsp) close to margin of apical orifice, large and deeply grooved in the middle to its left-apical side, bsp divided into two branches, left branch always narrow and simple, while right branch much thicker and showing morphology diversity among different species: dorsal surface prominent forming a hooked tubercle (Figs 19–21, 25), apex extended and bent forming a spiral-shaped structure (Figs 22, 26), or U-shaped with dorsal surface sinuate (Figs 23, 24, 27, 28); gonopore lobe long and directed to the base of endophallus. Female genitalia: Gonocoxite 2 of ovipositor almost identical across in species: falciform in ventral view, length three times the basal width; outer margin with one or two minute ensiform setae, inner margin without ensiform seta; apex rounded in lateral view, with two nematiform setae in a groove (Fig. 40). Spermatheca with seminal canal and receptaculum hardly differentiated; receptaculum straight and digitate, surface smooth; seminal canal a little slenderer than receptaculum, two to three times length of receptaculum; spermathecal gland inserted on base of receptaculum (Fig. 41).

The *Pterostichuspulcher* species group contains the following seven species, all distributed in the Liangshan Yi Autonomous Prefecture in Sichuan province of China (Fig. 42):

Pterostichus (Orientostichus) pulcher Sciaky & Allegro, 2013 (Meigu, E’bian);

Pterostichus (Orientostichus) pemphis sp. nov. (Puge, Zhaojue, Butuo);

Pterostichus (Orientostichus) orbicollis sp. nov. (Huili);

Pterostichus (Orientostichus) leo sp. nov. (Jinyang);

Pterostichus (Orientostichus) liyuani sp. nov. (Puge, Zhaojue);

Pterostichus (Orientostichus) condylus sp. nov. (Mianning, Xide);

Pterostichus (Orientostichus) jialini sp. nov. (Huidong).

### ﻿Key to species of the Pterostichus (Orientostichus) pulcher species group

**Table:** 

1	Elytral interval 3 with 1 (sometimes 0 or 2) small discal pore, interval 5 without discal pore, all intervals regular	**other species of subgenus Orientostichus**
–	Elytral intervals 3 and 5 each with ≥ 3 deeply foveate discal pores, interrupting intervals and forming catenulate sculpturing (*P.pulcher* group)	**2**
2	Antennomere 3 with accessory setae in addition to setae of apical ring; pronotum almost circular, lateral margins evenly curved before posterior angles (Figs 7–9); endophallus with right branch of bsp prominent forming a hooked tubercle (Figs 19–21, 25)	**3**
–	Antennomere 3 without accessory setae, only with primary setae forming apical ring; pronotum subcordate, lateral margins gradually narrowed to posterior angles, straight or sinuate before posterior angles (Figs 10–12); endophallus with right branch of bsp not tuberculate (Figs 22–24, 26–28)	**5**
3	Pronotal basal foveae with outer groove well-defined, ~ 2/3 of the length of inner groove (Figs 7, 8); male sternite VII with a small smooth tubercle (Figs 34, 35); apical lamella of aedeagus wide and short (LL/LW < 1.6), apex not turned upward (Figs 13, 14)	**4**
–	Pronotal basal foveae with outer groove indistinct, shorter than 1/2 of inner groove (Fig. 9); male sternite VII smooth, without sexual modification; apical lamella of aedeagus subuliform, very slender (LL/LW > 2.4) with apex strongly turned upward (Fig. 15)	***P.leo* sp. nov.**
4	Pronotum with ≥ 2 mid-lateral setae; posterior angles rounded, with an additional prominent denticle (Fig. 7); apical lamella of aedeagus longer (LL/LW=1.35–1.55) (Fig. 13)	***P.pemphis* sp. nov.**
–	Pronotum with 1 mid-lateral seta; posterior angles completely rounded, not dentate (Fig. 8); apical lamella of aedeagus shorter (LL/LW=1.1–1.25) (Fig. 14)	***P.orbicollis* sp. nov.**
5	Elytral interval 7 regular, without discal pore	**6**
–	Elytral interval 7 catenulate, interrupted by ≥ 5 large foveate discal pores	**7**
6	Pronotum with lateral margins hardly sinuate before posterior angles; pronotal disc with distinct transverse wrinkles (Fig. 10); endophallus with spiral-shaped bsp (Figs 22, 26)	***P.liyuani* sp. nov.**
–	Pronotum with lateral margins evidently sinuate before posterior angles; pronotal disc without or with very faint transverse wrinkles (Fig. 11); endophallus with U-shaped bsp (Figs 23, 27)	***P.condylus* sp. nov.**
7	Pronotum strongly narrowed to base (PBW/PW = 0.65–0.67); apex of posterior angles blunt and not projected; basal foveae depressed between inner and outer grooves (Fig. 12); male sternite VII asymmetrically modified, with 1 large tubercle and 2 small protuberances on its right basal side (Fig. 38)	***P.jialini* sp. nov.**
–	Pronotum moderately narrowed to base (PBW/PW=0.75–0.81); apex of posterior angles acute and projected out; basal foveae convex between inner and outer grooves (Fig. 32); male sternite VII symmetrically modified, with 1 smooth tubercle (Fig. 39)	***P.pulcher* Sciaky & Allegro**

## ﻿Descriptions

### ﻿ Pterostichus (Orientostichus) pemphis sp. nov.

Taxon classificationAnimaliaColeopteraCarabidae

2B37CF9B-BA75-5404-8D7E-727CE42E0AEB

https://zoobank.org/69D91D53-ECC3-4E35-B0FE-7CE16F72A5B6

**Figures 1–6.**
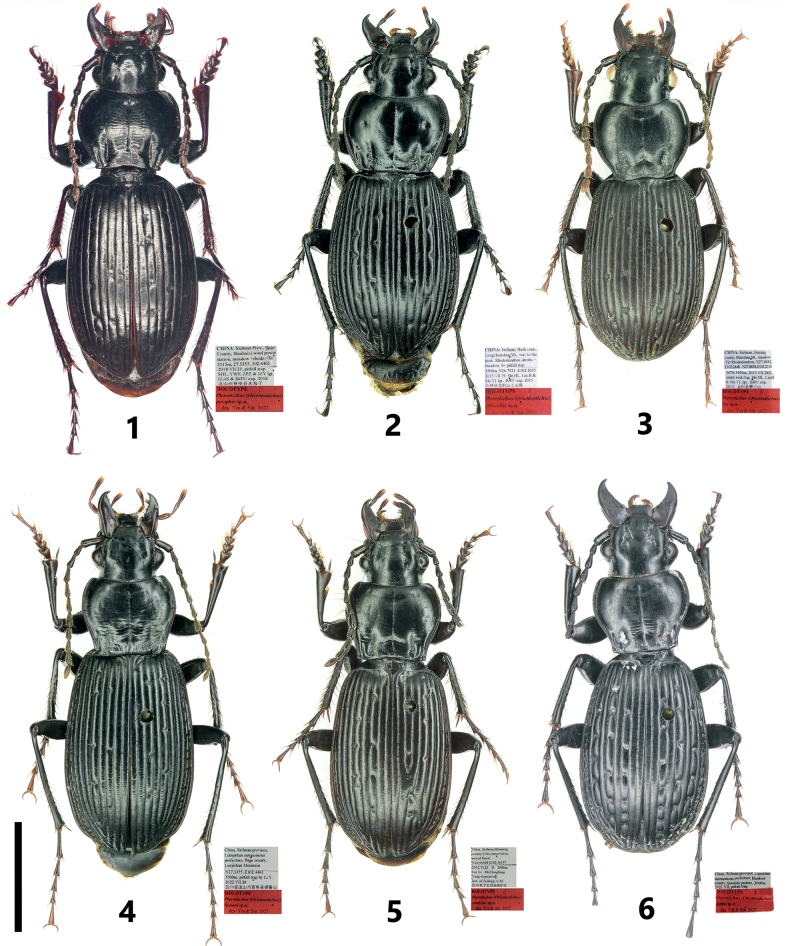
Habitus and labels of *Pterostichuspulcher* species group **1***P.pemphis* sp. nov., holotype **2***P.orbicollis* sp. nov., holotype **3***P.leo* sp. nov., holotype **4***P.liyuani* sp. nov., holotype **5***P.condylus* sp. nov., holotype **6***P.jialini* sp. nov., holotype. Scale bar: 5 mm.

**Figures 7–12.**
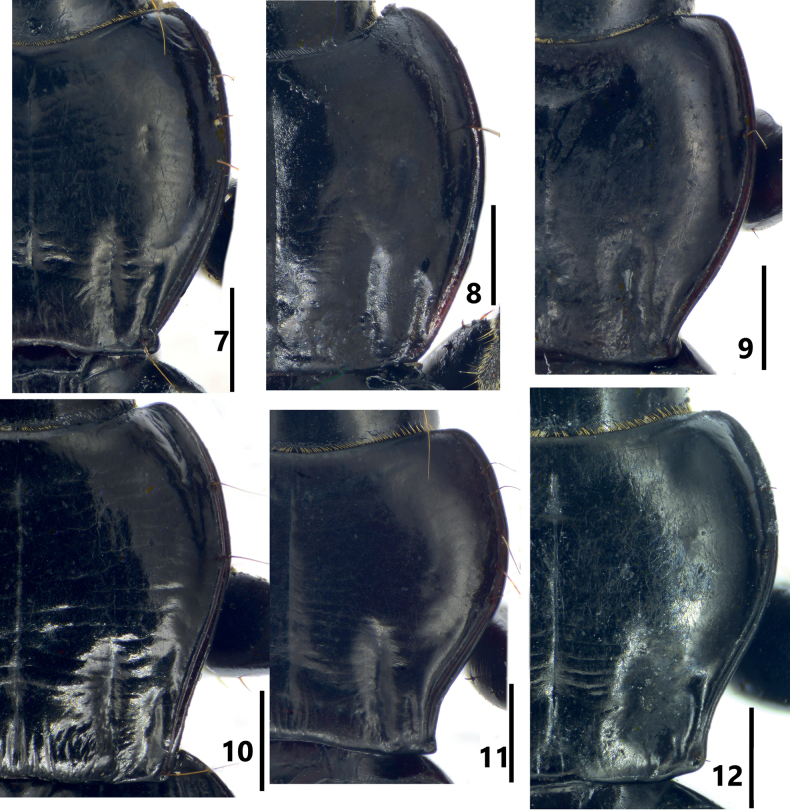
Pronota of *Pterostichuspulcher* species group **7***P.pemphis* sp. nov., holotype **8***P.orbicollis* sp. nov., holotype **9***P.leo* sp. nov., holotype **10***P.liyuani* sp. nov., holotype **11***P.condylus* sp. nov., holotype **12***P.jialini* sp. nov., holotype. Scale bars: 1 mm.

**Figures 13–18.**
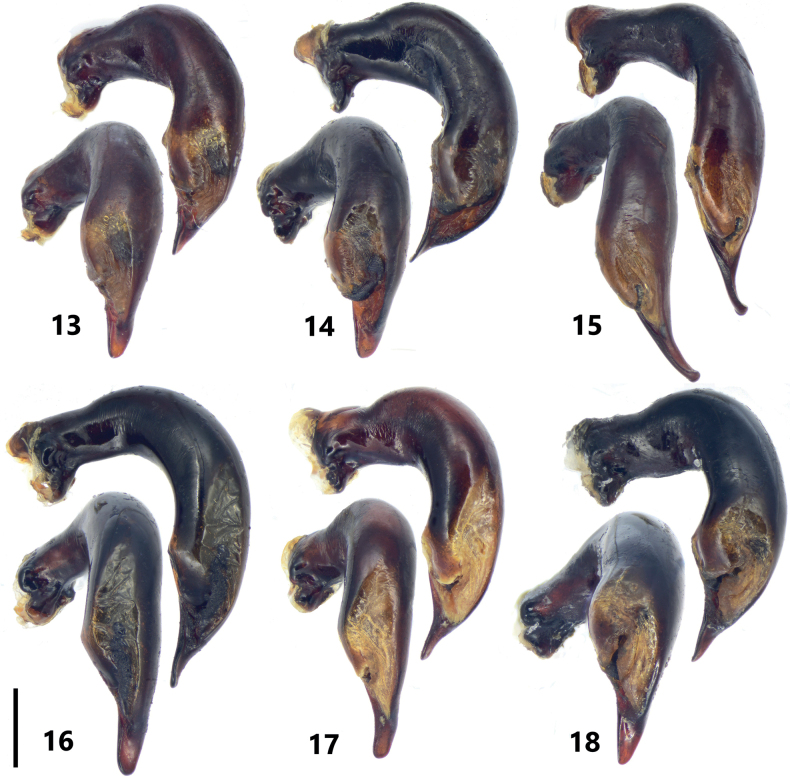
Male genitalia of *Pterostichuspulcher* species group, left lateral view and dorsal view of median lobe of aedeagus **13***P.pemphis* sp. nov., holotype **14***P.orbicollis* sp. nov., holotype **15***P.leo* sp. nov., holotype **16***P.liyuani* sp. nov., holotype **17***P.condylus* sp. nov., holotype **18***P.jialini* sp. nov., holotype. Scale bar: 1 mm.

**Figures 19–24.**
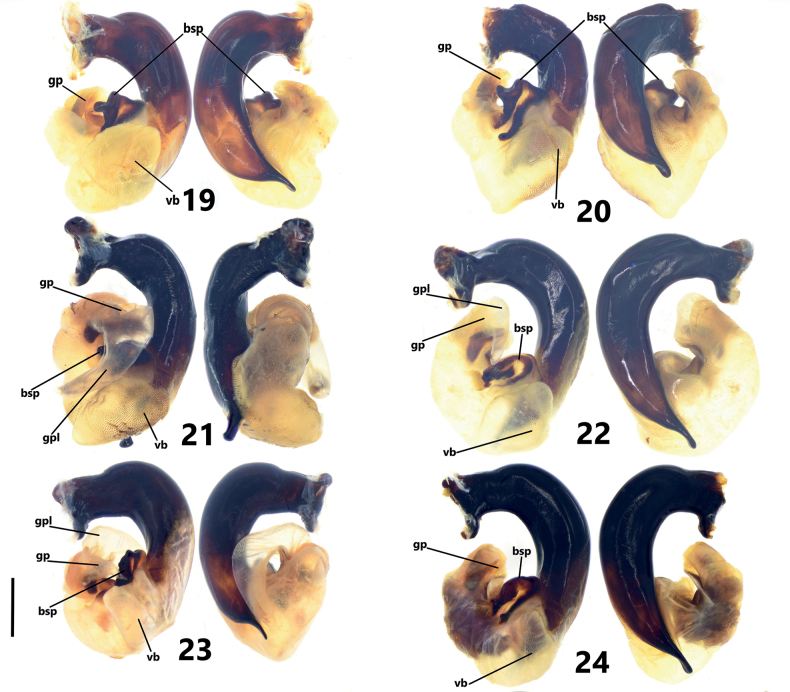
Endophallus of *Pterostichuspulcher* species group, left lateral view and right lateral view **19***P.pemphis* sp. nov., paratype from Zhaojue county **20***P.orbicollis* sp. nov., paratype from Longzhoushan mountain, Huili county **21***P.leo* sp. nov., paratype from Shizishan mountain, Jinyang county **22***P.liyuani* sp. nov., paratype from Luojishan mountain, Puge county **23***P.condylus* sp. nov., paratype from Mianning county **24***P.pulcher* Sciaky & Allegro, paratype from Yizi Yakou, E’bian county to Meigu county. Scale bar: 1 mm. Abbreviations: gp: gonopore, gpl: gonopore lobe, bsp: basal sclerotized projection, vb: ventral basal lobe.

Figs 1 , 7 , 13 , 19 , 34 , 40 , 41 Chinese vernacular name: 泡通缘步甲

#### Type locality.

China, Sichuan province: Liangshan Yi Autonomous Prefecture, Puge county, Shuihaizi wind power station (27.33N, 102.45E, alt 3515 m).

#### Type material.

***Holotype***: ♂: “China: Sichuan prov., Puge County, Shuihaizi wind power station, meadow + rhodo. + fir. 3515 m, N27.3355, E102.4461”, “2018.VII.21, pitfall trap. Shi HL, Yan WF, Zhu PZ & Jiang ZY lgt., IZAS & BJFU exp. 2018”, “HOLOTYPE ♂ Pterostichus (Orientostichus) pemphis sp. nov. det. Yin & Shi, 2022” [red label]; ***Paratypes*** (a total of 96 ♂ and 79 ♀): 19 ♂ and 24 ♀: the same data as holotype but labeled as paratype; 1 ♂ 2 ♀: “China: Sichuan prov., Butuo county, Juesa vill., Pukui mt., alpine meadow, 3248 m, N27.4233, E102.7604”, “2015.VII.26, pitfall trap, Shi HL, Liu B & Ma YL lgt., BJFU exp. 2015”, “PARATYPE of Pterostichus (Orientostichus) pemphis sp. nov. det. Yin & Shi, 2022” [red label]; 76 ♂ and 53 ♀ (CHYL): “China, Sichuan province, Liangshan autonomous prefecture, Zhaojue county, Jiefanggou town, 3000 m, 2022.VI, leg by Li Yuan”, “PARATYPE of Pterostichus (Orientostichus) pemphis sp. nov. det. Yin & Shi, 2022” [red label].

#### Diagnosis.

Elytral intervals 3 and 5 each with ≥ 3 large foveate discal pores, interval 7 without discal pore. Antennomere 3 with accessory setae. Pronotum nearly circular, evenly curved before posterior angles; posterior angles rounded, apex with an additional blunt denticle which distinctly prominent (Fig. 7); lateral margins with 2–4 mid-lateral setae. Male sternite VII with a well-defined smooth tubercle (Fig. 34). Apical lamella of aedeagus wide and short (LL/LW = 1.35–1.55), apex rounded (Fig. 13); endophallus with right branch of bsp tuberculate on dorsal surface (Fig. 19).

#### Comparison.

From the external features, *P.pemphis* sp. nov. is most similar to the following two new species, *P.leo* sp. nov. and *P.orbicollis* sp. nov. as all these three have accessory setae on antennomere 3, nearly circular pronotum, and dorsally tuberculate bsp on endophallus.

The present new species is different from *P.leo* sp. nov. by: (1) pronotum disc with distinct transverse wrinkles, but at most with very faint wrinkles in the latter species; (2) pronotal lateral margins evenly arched before posterior angles, posterior angles with a distinctly prominent denticle (Fig. 7), but in the latter species, pronotal lateral margins slightly sinuate before posterior angles, posterior angles with a smaller denticle (Fig. 9); (3) lateral margins with ≥ 2 mid-lateral setae, but with only one mid-lateral seta in the latter species; (4) male sternite VII with a smooth tubercle, but not modified in the latter species; (5) apical lamella of male genitalia short and straight, but strongly elongate and turned upward in the latter species.

Compared to *P.orbicollis* sp. nov., *P.pemphis* sp. nov. is different in: (1) posterior angles with a distinctly prominent denticle (Fig. 7), but not dentate in *P.orbicollis* (Fig. 8); (2) lateral margins with ≥ 2 mid-lateral setae, but with only one such seta in the latter species; (3) apical lamella of aedeagus a little longer than in *P.orbicollis* (LL/LW = 1.35–1.55 vs 1.1–1.25).

The present new species cohabitates with *P.liyuani* sp. nov. in Shuihaizi of Puge county. These two species can be easily distinguished by the differences on the shape of pronotum, the chaetotaxy on antennomere 3, and the sexual modification on male sternite VII (*P.pemphis* sp. nov. with a larger tubercle).

#### Description.

BL = 14.5–18.2 mm, BW = 5.0–6.0 mm, dorsal surface and appendages black, elytra often a little reddish brown. Antennomere 3 with accessory setae on apical 2/3 in addition to the primary setae forming apical ring. Pronotum nearly circular, PW/PL = 1.20–1.24, widest near anterior 1/3; anterior margin wider than posterior margin; strongly narrowed to base (PBW/PW = 0.61–0.65); lateral margins evenly arched from anterior angles to posterior angles, not sinuate before posterior angles; posterior angles rounded, apex with an additional blunt denticle which distinctly prominent; lateral margins with 2–4 mid-lateral setae near maximum width; basal foveae impunctate, inner and outer grooves straight, partly fused at base, outer groove a little shorter than inner one, area between them depressed (Fig. 7); disc with fine transverse wrinkles aside median line. Elytra oblong; parascutellar pore usually absent, but present in some specimens from Zhaojue county; intervals 3 and 5 each with ≥ 3 large foveate discal pores; interval 7 without discal pore. Male sternite VII with well-defined smooth tubercle on middle (Fig. 34). Median lobe of aedeagus stout, strongly curved near basal 1/3; apical lamella gradually narrowed and deflected ventrally; apical lamella slightly twisted longitudinally, relatively short (LL/LW = 1.35–1.55), apex rounded (Fig. 13). Endophallus long, straightly directed ventrally, gonopore opened to ventral-basal direction of aedeagus; vb distinct, oblate, rounded at both apexes; bsp deeply grooved, nearly U-shaped, dorsal surface of right branch folded and strongly hooked forming a tubercle (Fig. 19).

#### Distribution.

This species is relatively widely distributed from Puge county to Zhaojue county, in Liangshan Yi autonomous prefecture (Fig. 42).

#### Etymology.

The scientific name of the new species is derived from the Greek root *pemph*-, meaning blister, referring to the bump-shaped elytral intervals 3 and 5 of the new species.

### ﻿ Pterostichus (Orientostichus) orbicollis sp. nov.

Taxon classificationAnimaliaColeopteraCarabidae

8963CB2D-CCD2-57FB-9335-39D13C231207

https://zoobank.org/90A3FB24-DEF5-4244-A000-004B432F6D12

**Figures 25–30.**
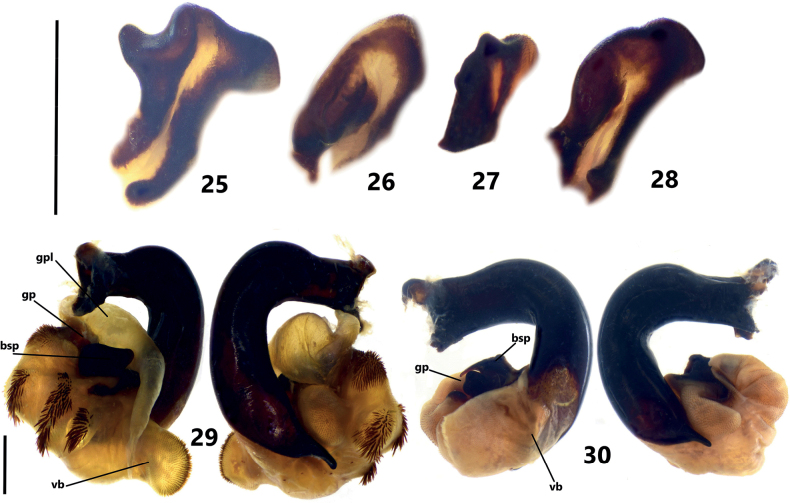
**25–28** endophallus bsp of *Pterostichuspulcher* species group, left lateral view **25***P.orbicollis* sp. nov., paratype from Longzhoushan mountain, Huili county **26***P.liyuani* sp. nov., paratype from Luojishan mountain, Puge county **27***P.condylus* sp. nov., paratype from Mianning county **28***P.pulcher* Sciaky & Allegro, paratype from Yizi Yakou, E’bian county to Meigu county **29, 30** Endophallus of Pterostichus (Orientostichus) spp., left lateral view and right lateral view **29***P.prattii* Bates, 1890, a male from Sanming city, Fujian province **30***P.curtatus* Fairmaire, a male from Heqing county, Yunnan province. Scale bars: 1 mm. Abbreviations: gp: gonopore, gpl: gonopore lobe, bsp: basal sclerotized projection, vb: ventral basal lobe.

Figs 2 , 8 , 14 , 20 , 25 , 35 Chinese vernacular name: 圜胸通缘步甲

#### Type locality.

China, Sichuan province: Liangshan Yi Autonomous Prefecture, Huili county, Longzhoushan mountain (26.79N, 102.20E, alt 3500 m).

#### Type material.

***Holotype***: ♂: “China: Sichuan, Huili county, Longzhoushan mt., way to the peak, Rhododendron shrubs + meadow, by pitfall trap”, “3500 m, N26.7921, E102.2035, 2015.VII.19, Shi HL, Liu B & Ma YL lgt., BJFU exp. 2015”, “HOLOTYPE ♂ Pterostichus (Orientostichus) orbicollis sp. nov. det. Yin & Shi, 2022” [red label]; ***Paratypes***: 6 ♂ and 16 ♀: the same data as holotype but labeled as paratypes.

#### Diagnosis.

Elytral intervals 3 and 5 each with ≥ 3 large foveate discal pores, interval 7 without discal pore. Antennomere 3 with accessory setae. Pronotum nearly circular; posterior angles completely rounded without denticle (Fig. 8); lateral margins with one mid-lateral seta. Male sternite VII with a well-defined smooth tubercle (Fig. 35). Apical lamella of aedeagus short and wide (LL/LW = 1.1–1.25), apex rounded (Fig. 14); endophallus with right branch of bsp tuberculate on dorsal surface (Figs 20, 25).

**Figures 31–33.**
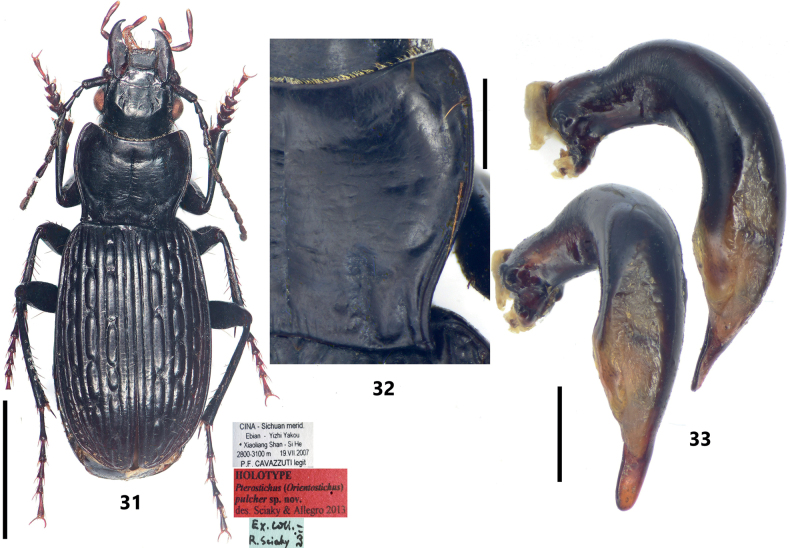
Holotype of *Pterostichuspulcher* Sciaky & Allegro, 2013 **31** habitus and labels **32** pronotum **33** male genitalia, left lateral view and dorsal view of median lobe of aedeagus. Scale bars: 5 mm (**31**); 1 mm (**32, 33**).

#### Comparison.

From the presence of accessory setae on antennomere 3 and relatively short apical lamella of male genitalia, *P.orbicollis* sp. nov. is most similar to *P.pemphis* sp. nov. But these two species can be easily distinguished by the differences on the number of pronotal mid-lateral setae and shape of pronotal posterior angles (details see Comparison under the latter species).

#### Description.

BL = 14.7–15.8 mm, BW = 5.5–6.0 mm, dorsal surface and appendages black, elytra sometimes a little reddish brown. Antennomere 3 with accessory setae on apical 2/3 in additional to the primary setae forming apical ring. Pronotum nearly circular, PW/PL = 1.30–1.34, widest near anterior 1/3; anterior margin wider than posterior margin; strongly narrowed to base (PBW/PW = 0.66–0.69); lateral margins evenly arched from anterior angles to posterior angles; posterior angles completely rounded, without a trace of denticle (Fig. 8); lateral margins with only one mid-lateral seta near maximum width; basal foveae impunctate, inner and outer grooves straight, partly fused at base, outer groove a little shorter than inner one, area between them slightly depressed; disc with fine transverse wrinkles aside median line. Elytra oblong, parascutellar pore absent; intervals 3 and 5 each with ≥ 3 large foveate discal pores; interval 7 without discal pore. Male sternite VII with well-defined tubercle on middle, small and smooth (Fig. 35). Median lobe of aedeagus stout, strongly curved near basal 1/3; apical lamella gradually narrowed and deflected ventrally; apical lamella slightly twisted longitudinally, relatively short (LL/LW = 1.1–1.25), with rounded apex (Fig. 14). Endophallus long, straightly directed ventrally, gonopore opened to ventral-basal direction of aedeagus; vb distinct, spherical, rounded at both apexes; bsp deeply grooved, nearly U-shaped, dorsal surface of right branch folded and strongly hooked forming a tubercle (Figs 20, 25).

#### Distribution.

This species was only found in the type locality, Longzhoushan mountain, Huili county, Liangshan Yi Autonomous Prefecture (Fig. 42).

#### Etymology.

The scientific name of the new species is composed of two Latin roots, *orbi*- meaning circular, and -*collis* meaning pronotum, referring to its completely rounded pronotum.

### ﻿ Pterostichus (Orientostichus) leo sp. nov.

Taxon classificationAnimaliaColeopteraCarabidae

F707D135-A354-5861-BBD5-48B6A22EE98A

https://zoobank.org/556A4863-E7B0-49D9-9374-03EE4EC36CE4

Figs 3 , 9 , 15 , 21 Chinese vernacular name: 狮通缘步甲

#### Type locality.

China, Sichuan province: Liangshan Yi Autonomous Prefecture, Jinyang county, Shizishan mountain (27.88N, 103.23E, alt 3470 m).

#### Type material.

***Holotype***: ♂: “China: Sichuan, Jinyang country, Shizishan mt., meadow /fir /rhododendron, N27.8882, E103.2448–N27.8838, E103.2310”, “3470–3493 m, 2015.VII.28, under rock/log., Shi HL, Liu B & Ma YL lgt. BJFU exp. 2015”, “HOLOTYPE ♂ Pterostichus (Orientostichus) leo sp. nov. det. Yin & Shi, 2022” [red label]; ***Paratypes***: 3 ♂ and 8 ♀: the same data as holotype but labeled as paratypes.

#### Diagnosis.

Elytral intervals 3 and 5 each with ≥ 3 large foveate discal pores, interval 7 without discal pore. Antennomere 3 with accessory setae. Pronotum nearly circular with slight sinuation before posterior angles, posterior angles with faintly pointed denticle (Fig. 9); lateral margins with one mid-lateral seta. Pronotal basal foveae with indistinct outer groove, shorter than 1/2 of inner groove. Male sternite VII without secondary sexual modification. Apical lamella of aedeagus subuliform, very slender with apex strongly turned upward, LL/LW = 2.4–2.65 (Fig. 15); endophallus with right branch of bsp tuberculate on dorsal surface (Fig. 21).

#### Comparison.

*P.leo* sp. nov. is peculiar among the *P.pulcher* species group for its unmodified male sternite VII and strongly upturned apical lamella of male genitalia, in comparing to other species which possess a tuberculate male sternite VII and conspicuously downward bent apical lamella. For the pronotum strongly narrowed to the base and the posterior angles more or less dentate, *P.leo* sp. nov. is most similar to *P.pemphis* sp. nov. But they can be readily distinguished by the differences on pronotum, male sternite VII and male genitalia (details listed in the comparison section of the latter species).

#### Description.

BL = 14.2–15.2 mm, BW = 5.3–5.6 mm, dorsal surface and appendages black, elytra often a little reddish brown. Antennomere 3 with accessory setae on apical 2/3 in additional to the primary setae forming apical ring. Pronotum nearly circular, PW/PL = 1.20–1.24, widest near anterior 1/3; anterior margin wider than posterior margin; strongly narrowed to base (PBW/PW = 0.63–0.68); lateral margins evenly arched from anterior angles to middle, slightly sinuate before posterior angles; posterior angles a little projected outward, forming a faintly denticle (Fig. 9); lateral margins with one mid-lateral seta near maximum width; basal foveae impunctate, inner and outer grooves straight, outer groove very shallow and shorter than 1/2 length of inner groove, area between them slightly depressed; disc without or with very faint transverse wrinkles aside median line. Elytra oblong; parascutellar pore absent; intervals 3 and 5 each with ≥ 3 foveate discal pores; interval 7 without discal pore. Male sternite VII without secondary sexual modification. Median lobe of aedeagus stout, strongly curved near basal 1/3; apical lamella of aedeagus subuliform, strongly elongate (LL/LW = 2.4–2.65) with apex conspicuously thickened and turned upward (Fig. 15). Endophallus long, straightly directed ventrally, gonopore opened to ventral-basal direction of aedeagus; vb distinct, spherical, rounded at both apexes; bsp deeply grooved, nearly U-shaped, dorsal surface of right branch folded and strongly hooked forming a tubercle (Fig. 21).

#### Distribution.

This species was only known from its type locality, Shizishan mountain, Jinyang county, Liangshan Yi Autonomous Prefecture (Fig. 42).

#### Etymology.

The scientific name of the new species derived from Latin, which means lion. It implies to the type locality of the new species, Shizishan Mt., which means “the mountain of lion” in Chinese.

### ﻿ Pterostichus (Orientostichus) liyuani sp. nov.

Taxon classificationAnimaliaColeopteraCarabidae

346E35B1-437A-5730-BA30-7B66F4002F56

https://zoobank.org/48C49F07-DDA3-418B-A284-EAA52596104F

Figs 4 , 10 , 16 , 22 , 26 , 36 Chinese vernacular name: 李圆通缘步甲

#### Type locality.

China, Sichuan province: Liangshan Yi Autonomous Prefecture, Puge county, Luojishan mountain (27.33N, 102.44E, alt 3500 m).

#### Type material.

***Holotype***: ♂: “China, Sichuan province, Liangshan autonomous prefecture, Puge county, Luojishan mountain”, “N27.3355, E102.4461, 3500 m, pitfall trap by Li Yuan, 2022.VII.26”, “HOLOTYPE ♂ Pterostichus (Orientostichus) liyuani sp. nov. det. Yin & Shi, 2022” [red label]; ***Paratypes*** (a total of 64 ♂ and 72 ♀): 18 ♂ and 15 ♀ (CHYL): the same data as holotype but labeled as paratype; 8 ♂ and 7 ♀: “China: Sichuan, Liangshan Dist., Puge county, Luojishan, tourist path, mixed forest, 2805 m, N27.58253 E102.39497”, “by pitfall trap, 2012.VI.10, Huang Hao lgt., Institute of Zoology, CAS”, “PARATYPE of Pterostichus (Orientostichus) liyuani sp. nov. det. Yin & Shi, 2022” [red label]; 6 ♂ and 29 ♀: “China: Sichuan prov., Puge County, Shuihaizi wind power station, meadow + rhodo + fir. 3515 m, N27.3355, E102.4461”, “2018.VII.21, pitfall trap. Shi HL, Yan WF, Zhu PZ & Jiang ZY lgt., IZAS & BJFU exp. 2018”, “PARATYPE of Pterostichus (Orientostichus) liyuani sp. nov. det. Yin & Shi, 2022” [red label]; 1 ♂: “China: Sichuan prov, Zhaojue county, Mufoshan mountain. peak. Rhododendron shrub + alpine meadow.”, “N28.0685, E102.7870, 3485 m. pitfall trap. 2015.VII.27. Shi HL, Liu B & Ma YL lgt.”, “PARATYPE of Pterostichus (Orientostichus) liyuani sp. nov. det. Yin & Shi, 2022” [red label]; 26 ♂ and 18 ♀: “China: Sichuan, Liangshan Dist., Puge county, Luojishan, near No. 1 glacial groove, mixed forest, 3470 m, N27.58445, E103.7913”, “by pitfall trap, 2012.VI.10, Huang Hao lgt., Institute of Zoology, CAS”, “PARATYPE of Pterostichus (Orientostichus) liyuani sp. nov. det. Yin & Shi, 2022” [red label]; 5 ♂ and 3 ♀: “China, Sichuan, Puge, Luojishan Mt., near ropeway upper station, 3595 m, N27.58108, E102.38012”, “2012.VI.10, pitfall trap, under rhododendra forest, Shi Hongliang & Liu Ye leg. Institute of Zoology, CAS.”, “PARATYPE of Pterostichus (Orientostichus) liyuani sp. nov. det. Yin & Shi, 2022” [red label].

#### Diagnosis.

Elytral intervals 3 and 5 each with ≥ 3 large foveate discal pores, interval 7 without discal pore. Antennomere 3 without accessory setae. Pronotum subcordate, with lateral margins nearly straight before posterior angles (Fig. 10); lateral margins with 2–4 mid-lateral setae; pronotal disc with shallow but distinct transverse wrinkles. Male sternite VII with a very small smooth tubercle (Fig. 36). Apical lamella of aedeagus short, apex rounded, LL/LW = 1.9–2.1 (Fig. 16); endophallus with spiral-shaped bsp (Figs 22, 26).

**Figures 34–39.**
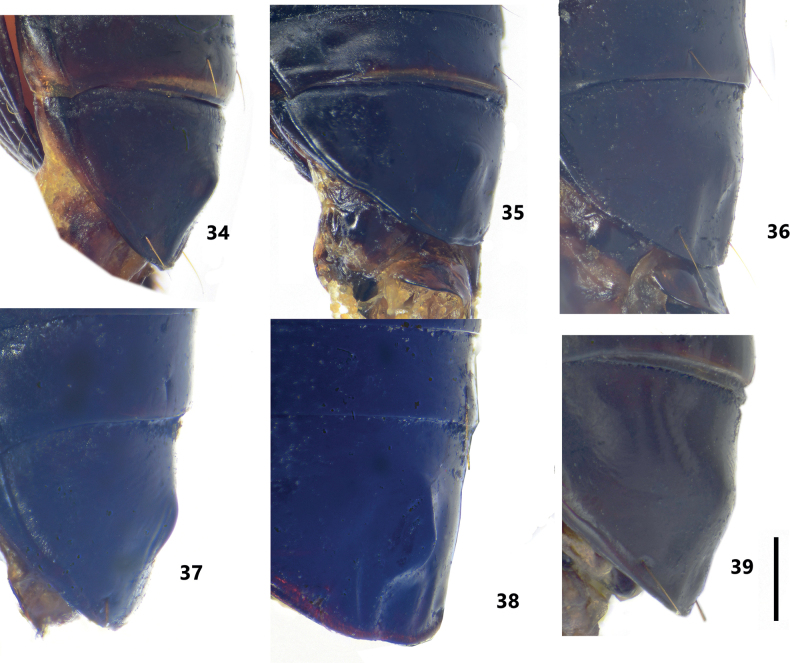
Male sternum VII of *Pterostichuspulcher* species group spp. **34***P.pemphis* sp. nov., holotype **35***P.orbicollis* sp. nov., holotype **36***P.liyuani* sp. nov., holotype **37***P.condylus* sp. nov., holotype **38***P.jialini* sp. nov., holotype **39***P.pulcher* Sciaky & Allegro, holotype. Scale bar: 1 mm.

#### Comparison.

From the external features, *P.liyuani* sp. nov. is most similar to another new species *P.condylus* sp. nov. These two species can be easily distinguished from *P.pemphis* sp. nov., *P.leo* sp. nov. and *P.orbicollis* sp. nov. by antennomere 3 without accessory setae and quite different pronotal shape, and from *P.pulcher* Sciaky & Allegro and *P.jialini* sp. nov. by interval 7 without discal pores.

Although these two new species are very similar to each other, *P.liyuani* sp. nov. is distinguishable from *P.condylus* sp. nov. by: (1) the sexual modification on male sternite VII is quite small and inconspicuous of *P.liyuani* sp. nov. (Fig. 36), while the latter species has larger and more well-defined tubercle on sternite VII (Fig. 37); (2) the pronotal disc is more evidently wrinkled in *P.liyuani* sp. nov., but the latter species at most has very faint wrinkles; (3) the pronotal lateral margin is hardly sinuate in *P.liyuani* sp. nov. (Fig. 10), but evidently sinuate in the latter species (Fig. 11); (4) comparing to *P.liyuani* sp. nov. (Fig. 16), in *P.condylus* sp. nov. (Fig. 17) the apical lamella of male genitalia is a little narrower, slightly narrowed near base and more truncated at apex; (5) the endophallus of *P.liyuani* sp. nov. has spiral shaped bsp with apex of right branch extended and bent reversely (Figs 22, 26) which is unique among this species group, while the latter species has a U-shaped bsp (Figs 23, 27).

**Figures 40, 41.**
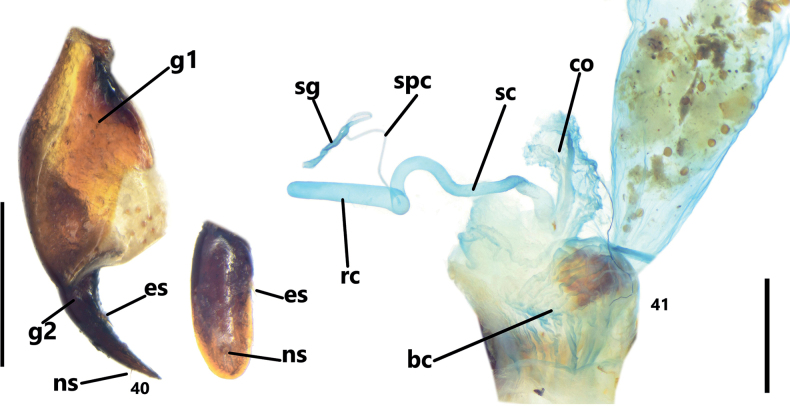
Female genitalia of Pterostichus (Orientostichus) pemphis sp. nov., paratype from Shuihaizi, Puge county **40** ventral view and inner lateral view of ovipositor. **41**. Female reproductive system. Scale bars: 0.5 mm (**40**); 1 mm (**41**). Abbreviations: g1: gonocoxite 1, g2: gonocoxite 2, es: ensiform setae, ns, nematiform setae, bc: bursa copulatrix, co: common oviduct, sc: seminal canal, rc: receptaculum, sg: spermathecal gland, spc: spermathecal canal.

#### Description.

BL = 14.7–18.5 mm, BW = 5.3–6.5 mm, dorsal surface and appendages black, elytra often a little reddish brown. Antennomere 3 without accessory setae, only with primary setae forming apical ring. Pronotum subcordate, PW/PL = 1.24–1.28, widest near anterior 1/3; anterior margin wider than posterior margin; slightly narrowed to base (PBW/PW = 0.71–0.75); lateral margins evenly arched from anterior angles to middle, hardly sinuate before posterior angles; posterior angles nearly rectangular, apex not dentate; lateral margins with 2–4 mid-lateral setae near maximum width; basal foveae often with slightly transverse wrinkles, inner and outer grooves straight, partly fused, outer groove slightly longer than 1/2 length of inner groove, area between them slightly depressed; disc with shallow but distinct transverse wrinkles aside median line. Elytra oblong; parascutellar pore usually absent, but present in one specimen from Zhaojue; intervals 3 and 5 each with ≥ 3 foveate discal pores; interval 7 without discal pore. Male sternite VII with an inconspicuous tubercle on middle, very small and smooth (Fig. 36). Median lobe of aedeagus stout, strongly curved near basal 1/3; apical lamella gradually dilated and deflected ventrally; apical lamella slightly twisted longitudinally, relatively long (LL/LW = 1.9–2.1), apex rounded (Fig. 16). Endophallus long, straightly directed ventrally, gonopore opened to ventral-basal direction of aedeagus; **vb** distinct, oblate, rounded at both apexes; bsp spiral-shaped, deeply grooved, apex of right branch strongly extended and bent reversely (Figs 22, 26).

#### Remarks.

The only examined specimen from Zhaojue county is different from other ones from Puge county for its elytral parascutellar pores present. It is interesting because most specimens of this species group have no parascutellar pores, but these pores are present only in some specimens collected from Zhaojue of two not closely related species, *P.liyuani* sp. nov. and *P.pemphis* sp. nov.

#### Distribution.

**Figure 42.**
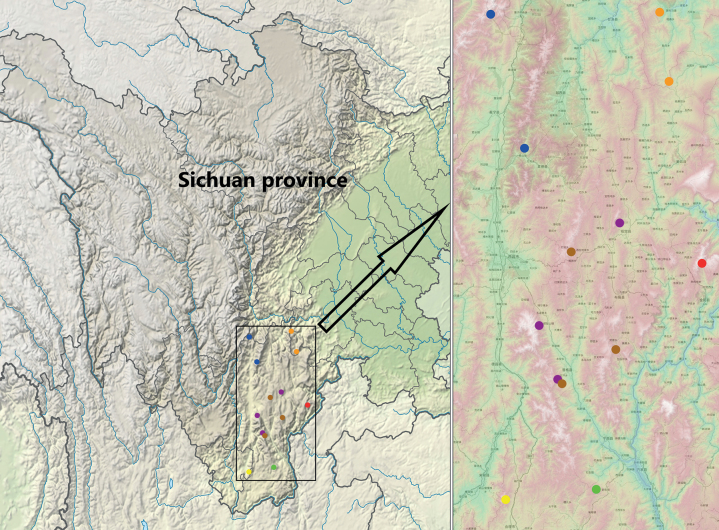
Distribution of the taxa of *Pterostichuspulcher* species group in Sichuan province, China. Brown: *P.pemphis* sp. nov. Yellow: *P.orbicollis* sp. nov. Red: *P.leo* sp. nov. Purple: *P.liyuani* sp. nov. Blue: *P.condylus* sp. nov. Green: *P.jialini* sp. nov. Orange: *P.pulcher* Sciaky & Allegro.

This species is known from two localities in Puge county and Zhaojue county, Liangshan Yi Autonomous Prefecture (Fig. 42).

#### Etymology.

This new species is named for Mr. Yuan Li, who contributed large number of specimens for the present study including many ones of this new species.

### ﻿ Pterostichus (Orientostichus) condylus sp. nov.

Taxon classificationAnimaliaColeopteraCarabidae

2D252550-2837-52B1-99C1-C2B15ED55936

https://zoobank.org/20D7AA84-D322-42BE-8503-7813663F7658

Figs 5 , 11 , 17 , 23 , 27 , 37 Chinese vernacular name: 肿通缘步甲

#### Type locality.

China, Sichuan province: Liangshan Yi Autonomous Prefecture, Mianning county, Yele. (28.96N, 102.16E, alt 2988 m).

#### Type material.

***Holotype***: ♂: “China: Sichuan, Mianning county, Yele conservation, mixed forest, N28.96508, E102.16137”, “2012.VI.22, day, 2988 m, Liu Ye, Shi Hongliang, Yang Ganyan leg. Inst. of Zoology, CAS.”, “HOLOTYPE ♂ Pterostichus (Orientostichus) condylus sp. nov. det. Yin & Shi, 2022” [red label]; ***Paratypes*** (a total of 4 ♂ and 6 ♀): 3 ♂ and 1 ♀: the same data as holotype but labeled as paratype; 1 ♂ and 2 ♀: “China: Sichuan, Xide county, Mianshan Village, Xiaoxiangling Mountain, alpine meadow, 3502 m, N28.4981, E102.3645”, “2018.VII.18, under dead log, Shi HL, Yan WF, Zhu PZ & Jiang ZY lgt. IZAS & BJFU exp of 2018”, “PARATYPE of Pterostichus (Orientostichus) condylus sp. nov. det. Yin & Shi, 2022” [red label]; 3 ♀: “Sichuan province, Liangshan Yi Autonomous Prefecture, Mianning county, Yele, 102.223059E, 28.918798N, 2549 m, Zhudong Liu, Zhiming Li and Pingzhou Zhu lgt., 2020.VIII.13”, “PARATYPE of Pterostichus (Orientostichus) condylus sp. nov. det. Yin & Shi, 2022” [red label].

#### Diagnosis.

Elytral intervals 3 and 5 each with ≥ 3 large foveate discal pores, interval 7 without discal pore. Antennomere 3 without accessory setae. Pronotum subcordate, lateral margins strongly sinuate before posterior angles, posterior angles slightly dentate (Fig. 11); pronotal disc without or with very faint transverse wrinkles; lateral margins with 2–4 mid-lateral setae. Male sternite VII with a small but distinct tubercle (Fig. 37). Apical lamella of aedeagus relatively slender, LL/LW = 1.8–2.0 (Fig. 17), apex rounded-truncated; endophallus with U-shaped bsp (Fig. 23).

#### Comparison.

In many aspects of morphology, *P.condylus* sp. nov. is most similar to *P.liyuani* sp. nov. The comparisons between these two species provided under *P.liyuani* sp. nov.

#### Description.

BL = 15.0–15.5 mm, BW = 5.2–5.5 mm, dorsal surface and appendages black. Antennomere 3 without accessory setae, only with primary setae forming apical ring. Pronotum subcordate, PW/PL = 1.35–1.39, widest near anterior 1/3; anterior margin slightly wider than posterior margin; moderately narrowed to base (PBW/PW = 0.69–0.73); lateral margins slightly arched from anterior angles to middle, and then strongly sinuate before posterior angles; posterior angles usually acute, slightly dentate and projected outward; lateral margins with 2–4 mid-lateral setae near maximum width; basal foveae deep and impunctate, inner and outer grooves nearly straight, outer groove distinctly shorter than 1/2 length of inner one (Fig. 11); disc without or with very faint transverse wrinkles aside median line. Elytra oblong; parascutellar pore absent; intervals 3 and 5 each with ≥ 3 foveate discal pores; interval 7 without discal pore. Male sternite VII with a tubercle on middle, small but well-defined (Fig. 37). Median lobe of aedeagus stout, strongly curved near basal 1/3; apical lamella gradually deflected ventrally; apical lamella slightly twisted longitudinally, relatively slender (LL/LW = 1.8–2.0), a little narrowed on basal 1/3 and gently bent leftward, apex rounded-truncated (Fig. 17). Endophallus long, straightly directed ventrally, gonopore opened to ventral-basal direction of aedeagus; vb quite small with rounded apex; bsp deeply grooved, U-shaped, left branch crenulate on dorsal margin (Figs 23, 27).

#### Distribution.

This species is known from two localities in Mianning and Xide counties, Liangshan Yi Autonomous Prefecture (Fig. 42).

#### Etymology.

The scientific name of the new species comes from a Greek root *condyl*- meaning lumps, referring to the well-defined tubercle on the male sternite VII of the new species.

### ﻿ Pterostichus (Orientostichus) jialini sp. nov.

Taxon classificationAnimaliaColeopteraCarabidae

F17393A4-0072-567C-8060-CCF5F507C7AB

https://zoobank.org/C0AC3F70-3137-4167-8308-44A1E4D1105E

Figs 6 , 12 , 18 , 38 Chinese vernacular name: 家麟通缘步甲

#### Type locality.

China, Sichuan province: Liangshan Yi Autonomous Prefecture, Huidong county, Jiamashi pasture (26.81N, 102.68E, alt 3000 m).

#### Type material.

***Holotype***: ♂: “China, Sichuan province, Liangshan autonomous prefecture, Huidong county, Jiamashi pasture, 3000 m, 2021.VII, pitfall trap”, “HOLOTYPE ♂ Pterostichus (Orientostichus) jialini sp. nov. det. Yin & Shi, 2022” [red label]. ***Paratype***: 1 ♂ (CYHL): the same data as holotype but labeled as paratype.

#### Diagnosis.

Elytra with several large foveate discal pores on intervals 3, 5, and 7, forming strong catenulate sculpturing. Antennomere 3 without accessory setae. Pronotum subcordate, strongly narrowed to base (PBW/PW = 0.65–0.67); posterior angles blunt and inconspicuously projected laterally; basal foveae depressed between inner and outer grooves which partly fused (Fig. 12). Sexual modification on sternite VII asymmetrical, with a large tubercle and two small protuberances on its right-basal side (Fig. 38). Apical lamella of male genitalia gradually narrowed to apex.

#### Comparison.

*P.jialini* sp. nov. is most similar to *P.pulcher* as both species differ from other five species of the *P.pulcher* species group in having large foveate discal pores on interval 7, forming catenulate sculpturing. Compared with *P.pulcher*, *P.jialini* sp. nov. is different in: (1) pronotum more strongly constricted to the base, with PBW/PW = 0.65–0.67 (vs PBW/PW = 0.75–0.81 in *P.pulcher*); (2) pronotum posterior angles blunt and inconspicuously projected laterally, while *P.pulcher* has posterior angles acute at apex and distinctly projected laterally; (3) pronotal basal foveae depressed between inner and outer grooves, but convex in *P.pulcher*; (4) male sternite VII with distinct and asymmetrical secondary sexual characters, while in *P.pulcher* male sternite VII shallowly and symmetrical tumid; (5) the apical lamella of male genitalia with its lateral margins convergent to apex, but in *P.pulcher*, the apical lamella with lateral margins subparallel to apex.

#### Description.

BL = 15.4–15.5 mm, BW = 5.7–5.8 mm, dorsal surface and appendages black, elytra with very faint metallic luster. Antennomere 3 without accessory setae, only with primary setae forming apical ring. Pronotum subcordate, PW/PL = 1.13–1.15, widest near middle; anterior margin slightly wider than posterior margin; strongly narrowed to base (PBW/PW = 0.65–0.67); lateral margins evenly arched from anterior angles to middle, distinctly sinuate before posterior angles; posterior angles rather blunt, apex rounded-obtuse, inconspicuously projected laterally; lateral margins with 2–4 mid-lateral setae near maximum width; basal foveae impunctate, depressed between inner and outer grooves, making them seems partly fused together (Fig. 12); disc with fine transverse wrinkles aside median line. Elytra oblong; parascutellar pore absent; intervals 3, 5, and 7 each with ≥ 3 large foveate discal pores, forming strong catenulate sculpturing. Male sternite VII with asymmetric modification, a large tubercle bending to right and two small protuberances on its right-basal side (Fig. 38). Median lobe of aedeagus stout, strongly curved near basal 1/3; apical lamella gradually deflected ventrally; apical lamella slightly twisted longitudinally, (LL/LW = 1.75–1.9), subtriangular with lateral margins convergent to apex, apex rounded (Fig. 18). Endophallus not studied due to restricted number of specimens.

#### Distribution.

This species is only known from its type locality in Huidong county, Liangshan Yi Autonomous Prefecture (Fig. 42).

#### Etymology.

The scientific name of the new species is dedicated to Mr. Jialin Tian, the collector of two type specimens.

### ﻿ Pterostichus (Orientostichus) pulcher

Taxon classificationAnimaliaColeopteraCarabidae

Sciaky & Allegro, 2013

36580511-724E-57F9-B82F-260D188B29F9

Figs 24 , 28 , 31–33 , 39 Chinese vernacular name: 俊通缘步甲

Pterostichus pulcher Sciaky & Allegro, 2013: 114. (holotype in IZAS; type locality: CHINA: Prov. Sichuan, Yizhi Yakou).

#### Material examined.

***Holotype***: ♂: “China, Sichuan merid. E’bian – Yizhi Yakou, Xiaoliang Shan – Si He. 2800–3100 m, 2007.VII.19, Cavazzuti P.F. leg.”, “2013 from Sciaky.”, “HOLOTYPE ♂ Pterostichus (Orientostichus) pulcher sp. nov. des. Sciaky & Allegro, 2013” [red label]. ***Paratypes***: 8 ♂: “China, Sichuan Prov., Yizi pass btw., Meigu county and E’bian county, mixed forest; N28.67477, E103.05248”, “2923 m; 2012.VI.15; by pitfall trap; Shi Hongliang & Liu Ye leg. Institute of Zoology, CAS.”, “PARATYPE of Pterostichus (Orientostichus) pulcher sp. nov. des. Sciaky & Allegro, 2013” [red label]. ***Non-type materials***: 3 ♂ and 6 ♀: “Sichuan province, Leshan city, e’bian Yi Autonomous county, Heizhugou No. 615 forestry centre, 103.055994E, 28.676587N, 2859 m, Zhudong Liu, Zhiming Li and Tao Li lgt., 2019.VII.22”; 18 ♂ and 9 ♀: “Sichuan province, Leshan city, e’bian Yi Autonomous county, Heizhugou No.615 forestry centre, 103.055994E, 28.676587N, 2859 m, Zhudong Liu, Zhiming Li and Pingzhou Zhu lgt., 2020.V.28”.

#### Diagnosis.

Elytra with several large foveate discal pores on intervals 3, 5, and 7, forming strong catenulate sculpturing. Antennomere 3 without accessory setae. Pronotum subcordate, slightly narrowed to base (PBW/PW = 0.75–0.81); lateral margins evidently sinuate before posterior angles, posterior angles acute at apex, distinctly projected laterally; lateral margins with 2–4 mid-lateral setae; pronotal disc with shallow transverse wrinkles; basal foveae with inner and outer grooves well-defined (Fig. 32), area between them strongly convex. Male sternite VII with single well-defined smooth tubercle (Fig. 39). Apical lamella of male genitalia elongate (LL/LW = 1.75–1.95), lateral margins subparallel to apex, apex rounded (Fig. 33); endophallus with U-shaped bsp, left branch evenly convex on dorsal margin (Figs 24, 28).

#### Comparison.

*P.pulcher* is most similar to *P.jialini* sp. nov. for their interval 7 with several foveate discal pores. This character clearly distinguishes them from the other five species in the species group. The comparisons between these two species were provided under *P.jialini* sp. nov.

#### Distribution.

This species has a narrow distribution range on the border of Meigu and E’bian counties, Liangshan Yi Autonomous Prefecture, but locally abundant (Fig. 42).

##### ﻿Species removed from subgenus Orientostichus Sciaky & Allegro

### ﻿ Synuchus nitidus reticulatus

Taxon classificationAnimaliaColeopteraCarabidae

Lindroth, 1956

E28029B6-0126-5FC4-80C0-329FDCD63B99

Figs 43–45

Synuchus nitidus reticulatus Lindroth, 1956: 501 (holotype in ZMC, type locality: China: Prov. Chekiang, Mokanshan).
Tritrichis chinensis Jedlička 1962: 308(type in NMPC; type locality: Kiukiang). Syn. nov.
Pterostichus (Steropanus) chinensis : Sciaky 1996: 430.
Pterostichus (Orientostichus) chinensis : Fedorenko 2018: 111.

#### Material examined.

***Holotype***: Holotype of *Tritrichischinensis* Jedlička, 1962, ♂ (NMPC) “Kiu-Kiang/China mer.”, “TYPUS” [red label], “Mus. Nat. Pragae/Inv. 26018” [orange label], “*Tritrichischinensis*/sp.n./det. Ing. Jedlička” [pink label]. ***Non-type materials***: more than 500 specimens from China (Gansu, Shaanxi, Henan, Anhui, Jiangsu, Zhejiang, Fujian, Jiangxi, Hunan, Hubei, Chongqing, Sichuan, Yunnan, Guizhou, and Guangxi provinces).

#### Distribution.

This subspecies is widely distributed in south China: Gansu*, Shaanxi*, Henan*, Anhui*, Jiangsu, Zhejiang, Fujian, Jiangxi*, Hunan*, Hubei, Chongqing*, Sichuan*, Yunnan*, Guizhou*, Guangxi* (based on our examined specimens, new province records in China are marked by asterisks). The distribution of HEB (Hebei) is most likely a misspelling of HUB (Hubei) in the Catalogue of Palaearctic Coleoptera (Hovorka 2017). The nominate subspecies *Synuchusnitidusnitidus* (Motschulsky, 1862) is distributed China (Liaoning), North Korea, South Korea, Russia (Sakhalin Island and Kuril Islands) and Japan.

#### Discussion.

**Figures 43–45.**
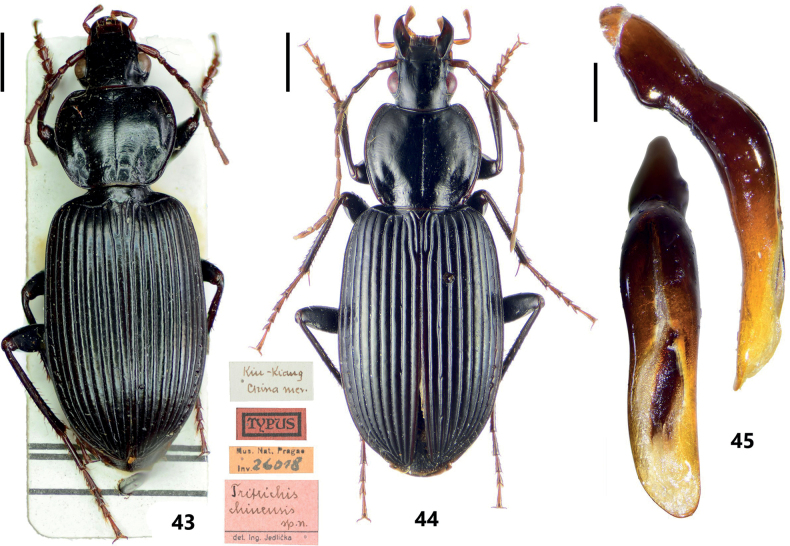
*Synuchusnitidusreticulatus* Lindroth, 1956 **43** habitus and labels of holotype of *Tritrichischinensis* Jedlička, 1962 **44, 45** a non-type male specimens from Guizhou, China **44** habitus **45** male genitalia, left lateral view and dorsal view of median lobe of aedeagus. Scale bars: 2 mm (**43, 44**); 0.5 mm (**45**).

*Tritrichischinensis* was described by Jedlička (1962). Subsequently, Sciaky (1996) treated these two genus-group names *Tritrichis* Andrewes and *Steropanus* Fairmaire as synonyms, and this species was assigned to the subgenus Steropanus of genus *Pterostichus*. Thereafter, based on the original description only, Fedorenko (2018) suggested the placement of *Pterostichuschinensis* (Jedlička) within the subgenus Orientostichus. One of us (HS) examined the holotype of *Tritrichischinensis* Jedlička in NMPC (Fig. 43) and discovered that the specimen belongs to the genus *Synuchus* as it has non-crossed elytral epipleuron, pectinate claws, broad terminal labial palpomere and a typical form of pronotum in *Synuchus*. After comparing many specimens (Fig. 44) collected from areas near the type locality, Jiangxi, we found that the holotype of *Tritrichischinensis* Jedlička is identical to *Synuchusnitidusreticulatus* Lindroth, 1956 in many aspects of morphology, including the rounded shape of pronotum, the broadly securiform terminal labial palpomere, and the linear microsculpture on elytra. Although the male genitalia of the holotype was not studied, we are confident that *Tritrichischinensis* Jedlička is a junior synonym of *Synuchusnitidusreticulatus* Lindroth.

## Supplementary Material

XML Treatment for Orientostichus

XML Treatment for Pterostichus (Orientostichus) pemphis

XML Treatment for Pterostichus (Orientostichus) orbicollis

XML Treatment for Pterostichus (Orientostichus) leo

XML Treatment for Pterostichus (Orientostichus) liyuani

XML Treatment for Pterostichus (Orientostichus) condylus

XML Treatment for Pterostichus (Orientostichus) jialini

XML Treatment for Pterostichus (Orientostichus) pulcher

XML Treatment for Synuchus nitidus reticulatus

## ﻿Additional information

### Author ORCIDs

Wenqi Yin https://orcid.org/0000-0003-2903-1399

Pingzhou Zhu https://orcid.org/0000-0003-2139-6764

Hongliang Shi https://orcid.org/0000-0002-9989-5830

### Data availability

All of the data that support the findings of this study are available in the main text.
